# Atypical hemolytic and uremic syndrome due to C3 mutation in pancreatic islet transplantation: a case report

**DOI:** 10.1186/s12882-020-02062-7

**Published:** 2020-09-19

**Authors:** Thibault Bahougne, Jérome Olagne, Marion Munch, Laura Braun-Parvez, Marie-Pierrette Chenard, Véronique Frémeaux-Bacchi, Sophie Caillard, Philippe Baltzinger, Michel Greget, Laurence Kessler, Bruno Moulin

**Affiliations:** 1 Service d’Endocrinologie, Diabète et Nutrition, Hôpitaux Universitaires de Strasbourg, 1 Place de L’Hôpital, F-67000 Strasbourg, France; 2 Institut des Neurosciences Cellulaires et Intégratives, CNRS (UPR 3212), Strasbourg, France; 3 Service de Néphrologie et Transplantation, Hôpital Civil, Strasbourg, France; 4 Service d’Anatomie Pathologique, Hôpital de Hautepierre, Hôpitaux Universitaires de Strasbourg, Strasbourg, France; 5 Assistance Publique-Hôpitaux de Paris, Laboratoire d’Immunology, Hôpital Européen Georges Pompidou, Paris, France; 6 INSERM UMR S1138, Complément et Maladies, Centre de Recherche des Cordeliers, Paris, France; 7 Service de radiologie, Hôpital de Hautepierre, Hôpitaux Universitaires de Strasbourg, Strasbourg, France; 8 Inserm UMR 1260, Nanomédecine Régénérative, Fédération de médecine Translationnelle, Université de Strasbourg, Strasbourg, France

**Keywords:** Atypical hemolytic and uremic syndrome, Complement C3, Islets of Langerhans transplantation, Case report

## Abstract

**Background:**

We here report on the first observation of a C3 mutation that is related to atypical hemolytic and uremic syndrome (aHUS), which occurred in a pancreatic islet transplant patient. Immunosuppressive treatments, such as calcineurin inhibitors, have been linked to undesirable effects like nephrotoxicity.

**Case presentation:**

A 40-year-old man with brittle diabetes, who was included in the TRIMECO trial, became insulin-independent 2 months after pancreatic islet transplantation. About 15 months after islet transplantation, the patient exhibited acute kidney injury due to aHUS. Despite plasma exchange and eculizumab treatment, the patient developed end-stage renal disease.

A genetic workup identified a missense variant (p.R592Q) in the C3 gene. In vitro, this C3 variant had defective Factor I proteolytic activity with membrane proteins as cofactor proteins, which was thus classified as pathogenic. About 1 year after the aHUS episode, kidney transplantation was carried out under the protection of the specific anti-C5 monoclonal antibody eculizumab. The patient had normal kidney function, with preserved pancreatic islet function 4 years later.

**Conclusions:**

Pancreatic islet transplantation could have triggered this aHUS episode, but this link needs to be clarified. Although prophylactic eculizumab maintains kidney allograft function, its efficacy still needs to be studied in larger populations.

## Background

Pancreatic islet transplantation has become a new therapeutic option for patients with unstable Type 1 diabetes mellitus *(*i.e. impaired awareness of hypoglycemia with an extreme glycemic lability). Due to corticosteroid-free immunosuppressive regimens, the insulin-independence rate has increased by up to 40% in the last 10 years [1]. Although the use of calcineurin inhibitors (CNI) enables similarly satisfying immunosuppressive levels, it carries the risk of associated undesirable effects, such as nephrotoxicity. Thrombotic microangiopathy (TMA) is a classic renal complication that occurs after organ transplantation in CNI-treated patients. This condition is characterized by microangiopathic hemolytic anemia, thrombocytopenia, and renal failure, which clinically result in hemolytic and uremic syndrome (HUS). Nevertheless, this condition may be difficult to distinguish from CNI toxicity and other causes of HUS, such as complement-mediated atypical HUS (aHUS) [2] or infection [3]. However, aHUS is a rarer form of HUS (less than 5% of cases) [4], which is associated with genetic or acquired defects in the proteins that regulate the alternate complement pathway, as well as autoantibodies that neutralize these proteins’ function. Specific genetic susceptibility variants may be identified in up to 60% of symptomatic individuals, but this requires a triggering event to initiate a flare-up [5]. In the pre-eculizumab era, about 50% of patients with pathogenic variants developed end-stage renal disease (ESRD) or died within 1-year follow-up [5].

Here, we report the case of a pancreatic islet recipient who developed aHUS that was associated with a complement component 3 (C3) gene mutation, along with secondary ESRD, which resulted in preventive eculizumab treatment for renal transplantation.

## Case presentation

A 40-year-old man with unstable Type I diabetes mellitus (with positive anti-insulin antibodies) was followed-up in the diabetology department of Strasbourg University Hospital. The duration of his diabetes was 35 years, during which he had normal renal function, no hematuria, stable microalbuminuria, and no other diabetic complications. Pre-transplant tests revealed neither HLA antibodies nor positive EBV serology, with negative CMV serology, normal cardiovascular exploration, and hepatic morphology. Since he did experience recurrent severe hypoglycemic episodes, the patient was included in the TRIMECO trial (https://clinicaltrials.gov/ct2/show/NCT01148680). In this trial, the patient received two pancreatic islet allografts using a percutaneous transhepatic portal approach over a 3-month period, without any immediate complications. Immunosuppressive induction for the first procedure included anti-thymocyte globulin (Thymoglobulin®, Sanofi Genzyme) at 0.5–1.5 mg/Kg/day for the first 3 days and anti-TNF-alpha etanercept (Enbrel®, AMGEN) on Day 0, which was decreased to 25 mg on Days 3, 7, and 10. Maintenance immunosuppression was conducted using 1000 mg of mycophenolate mofetil (Cellcept®, Genentech) twice a day and a CNI, tacrolimus (Prograf®, Astellas), twice a day, depending on the residual plasma levels (objectives: 9–13 μg/L for the first 3 months, then 6–9 μg/L thereafter). Induction for the second procedure consisted of 20 mg of the interleukin 2-receptor antagonist basiliximab (Simulect®, Novartis) on Days 0 and 4, along with etanercept. The total number of islets that were injected was 950,000 IEQ (islet equivalents to 150 pancreatic islet diameter). Two months after the second procedure, the patient became insulin-independent with a fasting C-peptide level of 2.3 μg/L (0.77 nmol/L), fasting glycemia of 5.7 mmol/L, and HbA1c of 5.1% (32 mmol/mol). This resulted in the disappearance of his hypoglycemic episodes. About 4 and 8 months after the second procedure, the patient was hospitalized due to recurrent watery diarrhea with Stage 1 acute kidney injury (AKI) [6], without any signs of hematological TMA. All microbiological explorations (bacterial, virologic, and parasitological in blood and feces) were negative, and kidney function returned to the normal range after intravenous hydration. This digestive episode was possibly linked to an undesirable effect of either mycophenolate mofetil or tacrolimus. However, these drugs were not discontinued.

**Fig. 1:**
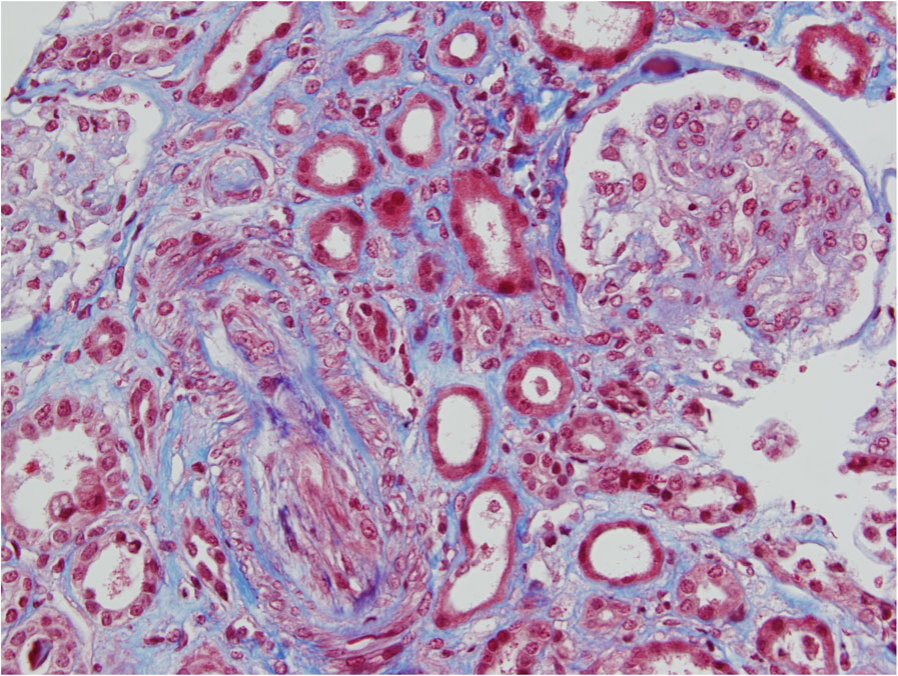
Histological renal biopsy analysis of the pancreatic islet-grafted patient with aHUS. Histological analysis abnormalities possibly related to a TMA process: (i) intra-arteriolar fibrinoid thrombi; (ii) nephroangiosclerosis lesions with intimal fibrinoid necrosis and foam endothelial cells; (iii) ischemic and sclerotic glomeruli (25%); (iv) intense tubulo-interstitial fibrosis. There was no thrombus detected in glomerular capillary, and no deposits detected in immunochemistry

About 15 months after the second procedure, the patient was readmitted to hospital for Stage 3 AKI and high blood pressure (200/100 mmHg). All blood and urine analysis results are presented in Table 1. The renal ultrasonography was normal, and the association of mechanical hemolytic anemia–thrombocytopenia and acute renal failure led us to suspect aHUS (Table 1). A hemodialysis session was initiated, along with plasma exchange (PEX; 60 mL/Kg) for 11 days. At this time, the first hypothesis established was direct endothelial toxicity due to overdosing of tacrolimus, in the context of dehydration from recurrent watery diarrhea, given that the trough tacrolimus level was at the upper limit of recommended targets. Since the suspicion was AKI/HUS induced by tacrolimus, the patient was first switched to ciclosporin (Neoral®, Novartis). He was subsequently switched to everolimus (Certican®, Novartis, anti m-TOR) after histological analysis of the renal biopsy results favored a typical TMA process (Fig. 1). The poor response to PEX and low C3 level led us to suspect aHUS, so eculizumab (Soliris®, ALEXION) was initiated. Eculizumab was started 18 days after AKI was diagnosed, at a weekly dose of 900 mg for the first 4 weeks. Thereafter, therapy was maintained at 1200 mg per week for a further 2 months [7]. At the same time, everolimus was similarly switched to cyclosporine. Although the thrombocytopenia resolved after 8 days of PEX, eculizumab administration was not followed by a recovery of renal function. During AKI, the islet graft remained functional. Despite the patient’s C-peptide level remaining uninterpretable in AKI, external insulin therapy was not deemed necessary to maintain normoglycemia.

**Table 1:** **Biological and immunological analysis at admission for acute renal injury**

**Blood analysis**	**Value**	**Normal range**
Creatinine (μmol/L)	680	53–97
Urea (mmol/L)	35	2.5–7
Hemoglobin (g/dL)	5.7	13–18
Schistocytes (%)	4	0
Platelets (count/mm3)	93.000	150.000–400.000
Haptoglobin (g/L)	< 0.08	0.72–1.92
LDH (IU/L) PT	765 198	120–246 100
**Urine stick**		
Blood	+++	–
Protein	+++	–
Leukocyte	–	–
Nitrite	–	–
**Urinalysis**		
Protein (g/L)	2.14	
**Immunological analysis**		
Total complement (IU/mL)	59	35–59
**C3 (g/L)**	**0.50**	**0.79–1.53**
C4 (g/L)	0.22	0.15–0.40
ANA	negative < 1/160	negative
ENA	negative	negative
Anti cardiolipids/β2GP1	negative	negative
PEP		
Albumin (g/L)	33	
Protein (g/L)	53	
ADAMTS 13	Activity > 20%	

*ADAMTS 13* A disintegrin and metalloprotease with thrombospondin Type 1 motifs, 13 member; *LDH* Lactate dehydrogenase; *PT* Prothrombin; *ANA* Antinuclear auto-antibodies; *ENA* Extractable nuclear antigen; *PEP* Protein electrophoresis

After receiving the patient’s written informed consent, blood samples were taken to screen for the six genes associated with aHUS. A rare nucleotide change (c.1775G > A) was detected in exon 14 of the C3 gene, resulting in an arginine-to-glutamine substitution in the C3 protein at position 570 (p.R592Q). This heterozygous variant, which has already been reported in patients diagnosed with aHUS [8], is an exposed amino acid located near the membrane cofactor protein (MCP) binding site. Functional analysis using a recombinant protein demonstrated decreased binding of the C3 variant with MCP, compared to the wild type, as well as a reduced rate of C3 cleavage by Factor I with MCP as the cofactor. This variant led to an indirect function gain, relative to complement activation, which explains the permanently low level of C3 in the patient’s plasma that was observed (Table 1). Genetic evaluations that were conducted on two sisters revealed the same C3 gene mutation, which contraindicated a related living kidney donation.

**Fig. 2:**
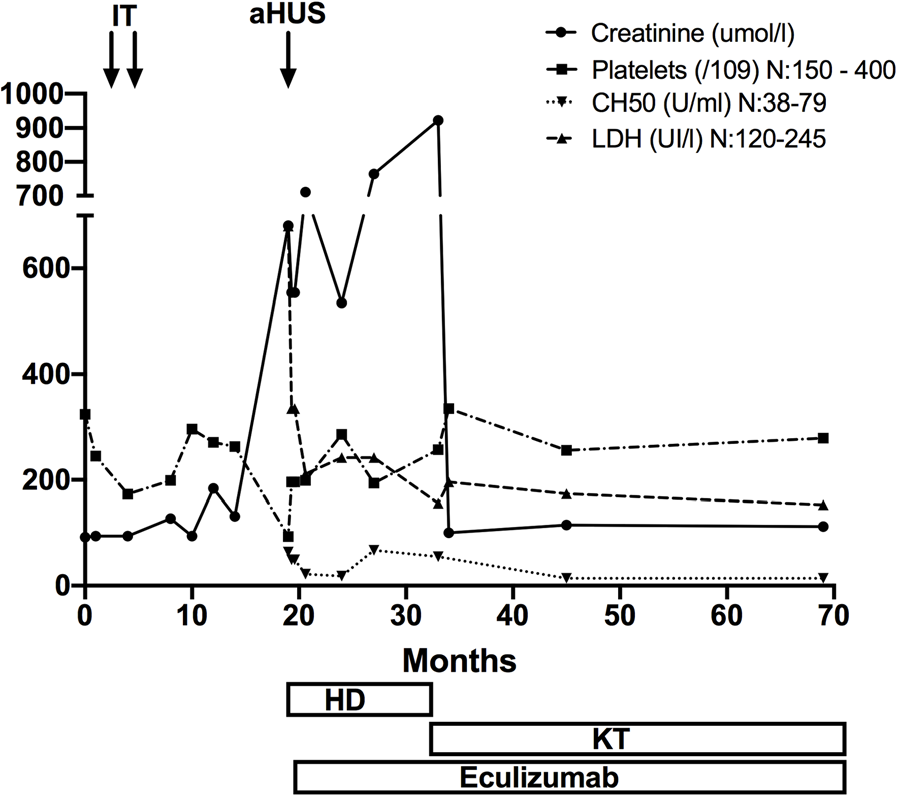
Biological monitoring of the pancreatic islet transplant (IT) patient 3 years following aHUS, treated using plasma exchange (PEX), eculizumab, hemodialysis (H)

The 40-year-old male patient eventually spent 1 year on hemodialysis treatment before undergoing brain-dead-donor kidney transplantation. There was no evidence for HLA immunization prior to transplantation, and preoperative HLA scoring identified four mismatches in HLA Class I, with none in HLA Class II. Cold ischemia time was 16 h. Immunosuppressive induction included basiliximab (Simulect®, Novartis), and eculizumab was continued at a dosage of 900 mg on Days 0, 1, 8, and 15, and then every other week at a dosage of 1200 mg to prevent aHUS relapse. Evidence of hematological TMA was absent in the immediate kidney transplantation follow-up. After kidney transplantation, the immunosuppressive regimen was modified to cyclosporine and mycophenolic acid (Myfortic®, Novartis). Three months after kidney transplantation, renal and pancreatic islet functions were considered excellent, with plasma creatinine levels at 94 μmol/L (1.06 mg/dL) and an HbA1c level of 5.5% (36.5 mmol/mol), without insulin therapy. Kidney function remained satisfactory 12 months after renal transplantation, with a plasma creatinine level of 87 μmol/L (1.0 mg/dL) without TMA lesions at the per-protocol kidney biopsy. Four years after renal transplantation, the patient is still treated with eculizumab, his plasma creatinine is 99 μmol/L, his low C3 level persists, and his pancreatic islet function is satisfactory. He has been insulin free for 5 years. The laboratory results pertaining to the 4-year follow-up are detailed in Fig. 2.

## Discussion and conclusions

Isolated pancreatic islet transplantation is not recommended for patients with Type I diabetes with kidney failure, due to CNI nephrotoxicity, which was initially suspected in our patient. However, double pancreatic islet and kidney transplantation in Type I diabetes patients with ESRD should be systematically considered, due to increased renal-graft survival, which is secondary to glycemic improvement.

HUS is categorized as typical when it is caused by the Shiga toxin-producing *Escherichia coli* infection, whereas atypical HUS is usually caused by uncontrolled complement activation. Additionally, HUS is categorized as secondary when it is accompanied by a coexisting disease. Genetic abnormalities are thought to act as predisposing factors for the development of the disease, and a trigger is required for the disease to manifest, such as an infection, kidney graft, or pregnancy. The common pathogenetic features in all HUS causes are simultaneous damage to endothelial cells, intravascular hemolysis, and activation of platelets. This leads to a pro-coagulative state, formation of microthrombi, and kidney damage. Yet, aHUS is a rare disorder, with an incidence of about 0.23–0.42 cases per million population [2]. The aHUS is characterized by a complement dysregulation in up to 50% of cases. Two types of mutation have been identified in aHUS patients. Loss-of-function mutations relate to the regulation of protein as complement Factor H (CFH), complement Factor I (CFI), MCP (or CD 46), diacylglycerol kinase epsilon (DGKe), and thrombomodulin genes [5]. Gain-of-function mutations relate to constitutive proteins of the C3 convertase alternative pathway, including C3 and complement Factor B (CFB). C3 is the principal protein of classical and alternative complement pathways, which are a natural defense system against invasive microbial infection. Maintenance of the complement system involves a balance between both activation and inhibition. Hereditary C3 deficiency is associated with severe recurrent infections and immune diseases. Between 5 and 10% of aHUS patients carry a heterozygous genetic abnormality in the C3 gene, which is classified as a gain-of-function pathogenic variant. Prior to the anti-C5 drug, the outcome was poor, with 60–70% of patients developing ESRD during the first few years. Compared to global cohorts, this clinical outcome did not differ from patients with a CFH mutation. Overall, more than 500 rare variants have been identified in aHUS, which have been registered in the new Database of Complement Gene Variants [9]. C3 mutations are mostly gain-of-function mutations of the C3 convertase, which are indirectly impaired through MCP regulation [10]. In our case, the variant R570Q (corresponding to p.592Q with the sequence of the peptide signal) was previously reported to induce a 70% decrease in C3/MCP binding. This induces cleavage resistance of the C3 convertase alternative pathway, which is normally induced by CFI [8]. The change from Arg to Gln in an exposed region is not conservative and may cause damaging structural effects. Screening of all genes identified as susceptibility factors for aHUS is mandatory in all patients that are diagnosed with aHUS without a coexisting disease (KDIGO, Goodship KI). Recently, Le Clech et al. [11] reported the low frequency of rare complement gene variants in secondary HUS patients (5%). In particular, after extra renal grafts, none of the nine patients with TMA carried a variant in the tested complement gene. Therefore, the screening of variants in complement genes does not appear to be relevant to HUS that is secondary to an extra renal graft. This should thus be performed in aHUS patients that do not have a coexisting disease. In this case, histology examination results are underwhelming, and the absence of glomerular thrombi may have been held against aHUS, in light of recent evidence, which further complicates the case. Low C3 levels strongly hint alternative pathway dysregulation, but only about 30–40% of patients with proven aHUS exhibit low C3 levels. In a cohort of 24 patients that were experiencing de novo aHUS following kidney transplantation, only six exhibited low C3 levels [12]. In our opinion, the prevailing argument remains that the severity of the AKI associated with hematological TMA signs is reason enough to perform genetic investigation.

To date, aHUS has never been described in pancreatic islet transplantation. In our patient, the TRIMECO protocol could be considered particularly aggressive for the endothelium, which acts as a trigger in the context of the patient’s C3 gene mutation, despite the Collaborative Islet Transplant Registry (CITR) not yet recording any cases of aHUS. The immunosuppressive regimen of the TRIMECO trial induces a form of endothelium toxicity that is probably comparable to that observed in organ transplantation, particularly in pancreas grafts. However, it is essential to consider the specificity of the vascularization of the islets during the graft, which probably leads to activation in both the donor and recipient endothelium. Indeed, the pro-inflammatory and pro-coagulative mechanisms of the instant blood mediated inflammatory reaction (IBMIR) have been well identified. These mechanisms lead to a complex process of revascularization of the islets in the donor and recipient endothelium [13], which can result in a specific susceptibility to aHUS. We cannot exclude the notion that this particular aggression in the endothelium represents a trigger in the context of the C3 gene mutation. To date, the context of islet transplantation and aHUS favored by C3 gene mutation has hardly been linked. However, this pathogenic variant has already been identified in post-kidney transplantation aHUS [12, 14]. With regards to the delay between aHUS and islet transplantation, de novo TMA has been reported to occur between 1 day and 23 months after kidney transplantation, which is probably favored by mutation in the CFH and CFI genes [12].

Despite a lack of direct comparison between PEX and eculizumab, evidence suggests that eculizumab is far superior. In this case, PEX was only initially used because secondary HUS (including CNI toxicity) was initially suspected. Conversely, as the efficacy of eculizumab is time sensitive, plasmapheresis may have needlessly delayed the eculizumab initiation, even though there was indirect evidence for aHUS (low levels of C3). This thereby contributed to the development of ESRD [15, 16].

The relapse risk of aHUS after kidney transplantation is considered high [17], with a recurrence of aHUS in kidney grafts occurring in 60–80% of patients. It has been demonstrated that patients with complement factor mutations exhibit a three-fold increase in post-transplant aHUS recurrence, compared to aHUS patients without mutations [18, 19], in patients with CAP dysregulation. Hence, without preventive treatment, kidney transplantation in the aHUS context is compromised by the risk of recurrence. Eculizumab, a human anti-C5 monoclonal antibody, represents the new treatment line for acute episodes and recurrence prevention in grafts [20]. Since a first publication in 2009 [21], further eculizumab studies have revealed a 70% complete response rate in terms of hematologic, renal, and quality-of-life improvement, as well as dialysis discontinuation and transplant protection [7, 22].

This case of a patient with a C3 gain-of-function mutation highlights the diagnostic challenge represented by TMA in the atypical context of islet transplantation. Calcineurin inhibitors that are associated with TMA must be taken into account after exclusion of other causes of HUS, especially using a genetic screening of potential complement mutations. Discriminating between secondary HUS and aHUS is exceedingly challenging, but clinicians may find solace in looking for clues such as lowered C3, the presence of glomerular thrombi on biopsy, or reduced levels of CFI, CFH, or MCP. They may also resort to more empirical decision-making, such as weighing the benefits of eculizumab therapy in the face of persistent AKI. Pancreatic islet transplantation, specifically the immunosuppressive regimen of the TRIMECO trial and IBMIR, could have triggered this aHUS episode, but this connection needs to be further explored. Since preemptive use of eculizumab in the setting of kidney transplantation and at-risk pathogenic variants (notably, C3 gain-of-function variants) has been advocated in expert reviews and guidelines [23, 24], this has gradually superseded historical aHUS treatment, which was liver transplantation [25]. However, issues concerning the duration of treatment currently remain unresolved. The context of combined transplantation, ongoing CNI therapy, and the nature of the pathogenic variant inform our assertion that eculizumab should be continued as long as either graft is functional.

## Data Availability

The datasets used or analyzed during the current study are available from the corresponding author on reasonable request.

## Abbreviations

aHUS: Atypical hemolytic uremic syndrome
AKI: Acute kidney injury
CFB: Complement factor B
CFH: Complement factor H
CFHR5: Complement factor H-related protein 5
CFI: Complement factor I
CNI: Calcineurin inhibitors
GKe: Diacylglycerol kinase epsilon
ESRD: End-stage renal disease
IEQ: Islet equivalents
MCP: Membrane cofactor protein
PEX: Plasma exchange
TMA: Thrombotic microangiopathy
TRIMECO: Trial comparing metabolic efficiency of islet graft to intensive insulin therapy for the treatment of type 1 diabetes

## References

[1] Lablanche S, Vantyghem M-C, Kessler L, Wojtusciszyn A, Borot S, Thivolet C (2018). Islet transplantation versus insulin therapy in patients with type 1 diabetes with severe hypoglycaemia or poorly controlled glycaemia after kidney transplantation (TRIMECO): a multicentre, randomised controlled trial. Lancet Diabetes Endocrinol.

[2] Fakhouri F, Zuber J, Frémeaux-Bacchi V, Loirat C (2017). Haemolytic uraemic syndrome. Lancet (London, England).

[3] George JN, Nester CM (2014). Syndromes of thrombotic Microangiopathy. N Engl J Med.

[4] Bayer G, von Tokarski F, Thoreau B, Bauvois A, Barbet C, Cloarec S (2019). Etiology and outcomes of thrombotic Microangiopathies. Clin J Am Soc Nephrol.

[5] Fremeaux-Bacchi V, Fakhouri F, Garnier A, Bienaimé F, Dragon-Durey M-A, Ngo S (2013). Genetics and outcome of atypical hemolytic uremic syndrome: a nationwide French series comparing children and adults. Clin J Am Soc Nephrol.

[6] Khwaja A (2012). KDIGO clinical practice guidelines for acute kidney injury. Nephron.

[7] Fakhouri F, Hourmant M, Campistol JM, Cataland SR, Espinosa M, Gaber AO, et al. Terminal complement inhibitor Eculizumab in adult patients with atypical hemolytic uremic syndrome: a single-arm, open-label trial. Am J Kidney Dis. 2016;16:84–93. 10.1053/j.ajkd.2015.12.034 27012908

[8] Frémeaux-Bacchi V, Miller EC, Liszewski MK, Strain L, Blouin J, Brown AL (2008). Mutations in complement C3 predispose to development of atypical hemolytic uremic syndrome. Blood.

[9] Osborne AJ, Breno M, Borsa NG, Bu F, Frémeaux-Bacchi V, Gale DP (2018). Statistical validation of rare complement variants provides insights into the molecular basis of atypical hemolytic uremic syndrome and C3 Glomerulopathy. J Immunol.

[10] Roumenina LT, Frimat M, Miller EC, Provot F, Dragon-Durey M-A, Bordereau P (2012). A prevalent C3 mutation in aHUS patients causes a direct C3 convertase gain of function. Blood.

[11] Le Clech A, Simon-Tillaux N, Provôt F, Delmas Y, Vieira-Martins P, Limou S (2019). Atypical and secondary hemolytic uremic syndromes have a distinct presentation and no common genetic risk factors. Kidney Int.

[12] Le Quintrec M, Lionet A, Kamar N, Karras A, Barbier S, Buchler M (2008). Complement mutation-associated de novo thrombotic microangiopathy following kidney transplantation. Am J Transplant.

[13] Chen CC, Pouliquen E, Broisat A, Andreata F, Racapé M, Bruneval P (2018). Endothelial chimerism and vascular sequestration protect pancreatic islet grafts from antibody-mediated rejection. J Clin Invest.

[14] Milan Manani S, Virzì GM, Giuliani A, Clementi A, Brocca A, Dissegna D (2017). Hemolytic uremic syndrome and kidney transplantation: a case series and review of the literature. Nephron.

[15] Vande WJ, Delmas Y, Ardissino G, Wang J, Kincaid JF, Haller H (2017). improved renal recovery in patients with atypical hemolytic uremic syndrome following rapid initiation of eculizumab treatment. J Nephrol.

[16] Jamme M, Raimbourg Q, Chauveau D, Seguin A, Presne C, Perez P, et al. Predictive features of chronic kidney disease in atypical haemolytic uremic syndrome. PLoS One. 2017;12(5):e0177894. 10.1371/journal.pone.0177894 PMC5436831 28542627

[17] Krid S, Roumenina LT, Beury D, Charbit M, Boyer O, Frémeaux-Bacchi V (2012). Renal transplantation under prophylactic eculizumab in atypical hemolytic uremic syndrome with CFH/CFHR1 hybrid protein. Am J Transplant.

[18] Le Quintrec M, Zuber J, Moulin B, Kamar N, Jablonski M, Lionet A (2013). Complement genes strongly predict recurrence and graft outcome in adult renal transplant recipients with atypical hemolytic and uremic syndrome. Am J Transplant.

[19] Sellier-Leclerc A-L, Fremeaux-Bacchi V, Dragon-Durey M-A, Macher M-A, Niaudet P, Guest G (2007). Differential impact of complement mutations on clinical characteristics in atypical hemolytic uremic syndrome. J Am Soc Nephrol.

[20] Johnson S, Stojanovic J, Ariceta G, Bitzan M, Besbas N, Frieling M (2014). An audit analysis of a guideline for the investigation and initial therapy of diarrhea negative (atypical) hemolytic uremic syndrome. Pediatr Nephrol.

[21] Gruppo RA, Rother RP (2009). Eculizumab for congenital atypical hemolytic-uremic syndrome. N Engl J Med.

[22] Zuber J, Le Quintrec M, Krid S, Bertoye C, Gueutin V, Lahoche A (2012). Eculizumab for atypical hemolytic uremic syndrome recurrence in renal transplantation. Am J Transplant.

[23] Loirat C, Fakhouri F, Ariceta G, Besbas N, Bitzan M, Bjerre A (2016). An international consensus approach to the management of atypical hemolytic uremic syndrome in children. Pediatr Nephrol.

[24] Zuber J, Frimat M, Caillard S, Kamar N, Gatault P, Petitprez F, et al. Use of highly individualized complement blockade has revolutionized clinical outcomes after kidney transplantation and renal epidemiology of atypical hemolytic uremic syndrome. J Am Soc Nephrol. 2019;ASN.2019040331(12):2449–63. 10.1681/ASN.2019040331 PMC6900783 31575699

[25] Noris M, Remuzzi G (2010). Thrombotic Microangiopathy after kidney transplantation. Am J Transplant.

